# Comparing the effectiveness of different indicators of insulin resistance in predicting diabetes among adults with sarcopenia from the NHANES (1999–2018)

**DOI:** 10.1097/MD.0000000000045118

**Published:** 2025-10-10

**Authors:** Yingying Zhu, Jiabei He, Xin Li, Yunping Xu, Zongliang Yu, Kede Chi

**Affiliations:** a Department of Geriatric Medicine, Zhongshan Hospital of Traditional Chinese Medicine (The Tenth Clinical Medical College of Guangzhou University of Traditional Chinese Medicine), Zhong Shan, Guangdong Province, China; b National Clinical Research Center for Chinese Medicine Cardiology, Xiyuan Hospital, China Academy of Chinese Medical Sciences, Beijing, China; c Department of Orthopedics, Zhongshan Hospital of Traditional Chinese Medicine (The Tenth Clinical Medical College of Guangzhou University of Traditional Chinese Medicine), Zhong Shan, Guangdong Province, China.

**Keywords:** diabetes, indicators of insulin resistance, ROC, sarcopenia

## Abstract

The aim of this research is to investigate the correlation between indicators of various types of insulin resistance and the likelihood of developing diabetes in populations exhibiting sarcopenia. This cross-sectional study includes adults who participated in the National Health and Nutrition Examination Survey from 1999 to 2018. Insulin sensitivity indicators primarily include the IR metabolic score (METS-IR), triglyceride glucose index (TyG), triglyceride glucose index body mass index (TyG-BMI), triglyceride glucose index waist circumference (TyG-WC), and triglyceride to high-density lipoprotein cholesterol ratio for assessing insulin sensitivity. Muscle mass measurements are obtained using dual-energy x-ray absorptiometry. Employing various statistical techniques such as multiple logistic regression, analysis of receiver operating characteristic curves, subgroup analysis and interaction analysis will be conducted to assess the correlation between different indicators of insulin resistance and the likelihood of developing diabetes in populations affected by sarcopenia or those suspected to have sarcopenia. A total of 1632 adults aged over 18 were included in this study. It was observed that METS-IR, TyG index, TyG-BMI, TyG-WC, and triglyceride to high-density lipoprotein cholesterol were all positively associated with the risk of diabetes in populations with sarcopenia. The results remained consistent after adjusting for confounding factors. Receiver operating characteristic curve analysis demonstrated that TyG exhibited the most superior predictive efficacy for diabetes within this specific population cohort, particularly among males. Notably, a statistically significant distinction was observed in comparison to females (*P* < .05). The results of the subgroup analysis indicated that, across all examined subgroups, there was a positive correlation between the risk of diabetes and different indicators of insulin resistance in populations with sarcopenia. Our research findings suggest that METS-IR, TyG, TyG-BMI, TyG-WC, and TG/HDL are all positively associated with the incidence of diabetes in populations with sarcopenia. These biomarkers can be utilized for predicting the onset of diabetes in such populations. Among these biomarkers, TyG exhibits a higher predictive value for diabetes in this group, particularly among male individuals.

## 1. Introduction

As individuals age, muscle mass progressively declines, which is a hallmark feature of sarcopenia. Beginning at the age of 40, muscle mass decreases by approximately 8% per decade, and this rate accelerates to about 15% per decade after the age of 70.^[1]^ A global meta-analysis has demonstrated that the prevalence of sarcopenia ranges from 8% to 36% in individuals under 60 years old and from 10% to 27% in those aged 60 and older^.[2,3]^ Since 2016, the World Health Organization has officially classified sarcopenia as a disease within the International Classification of Diseases and Related Health Problems. With the ongoing progression of global aging, sarcopenia is anticipated to become a major public health challenge in the future.

Numerous clinical studies have indicated that individuals with sarcopenia are more likely to develop diabetes compared to those without sarcopenia, potentially due to insulin resistance.^[4–10]^ Skeletal muscle serves as one of the primary targets for insulin action, and insulin resistance may arise from a diminished responsiveness of insulin-sensitive tissues, such as skeletal muscle, to insulin. Moreover, insulin resistance can exacerbate sarcopenia by disrupting skeletal muscle metabolism and promoting excessive autophagy.

In clinical practice, the hyperinsulinemic-euglycemic clamp test and the homeostasis model assessment of insulin resistance are widely used to evaluate insulin resistance.^[11]^ Although the hyperinsulinemic-euglycemic clamp test is regarded as the gold standard for assessing peripheral insulin sensitivity, its complexity and time-consuming nature limit its practicality in routine clinical settings. Recently, several non-insulin-dependent indicators for assessing insulin sensitivity have been developed, providing a simpler and more reliable method for evaluating insulin resistance (IR) and metabolic syndrome. However, no systematic research has been conducted to assess the consistency and predictive power of these indicators in predicting diabetes risk among patients with sarcopenia. This study aims to evaluate the correlation between various insulin resistance indicators and diabetes risk in patients with sarcopenia and to compare the effectiveness of these indicators in predicting diabetes in this specific population.

## 2. Materials and methods

### 2.1. Study population

All data for this study were obtained from the National Health and Nutrition Examination Survey (NHANES) database spanning the years 1999 to 2018. The NHANES database encompasses cross-sectional surveys conducted biennially by the Centers for Disease Control and Prevention. The study protocol for the NHANES database was approved by the Ethics Review Committee of the National Center for Health Statistics, and participants provided informed consent through signed consent forms. Following NCHS policy, data from the NHANES database were acquired without direct participant contact, enabling their utilization in data analysis without further institutional ethics committee review. This study adhered to the Reporting of Observational Studies in Epidemiology guidelines.

**Figure 1.**
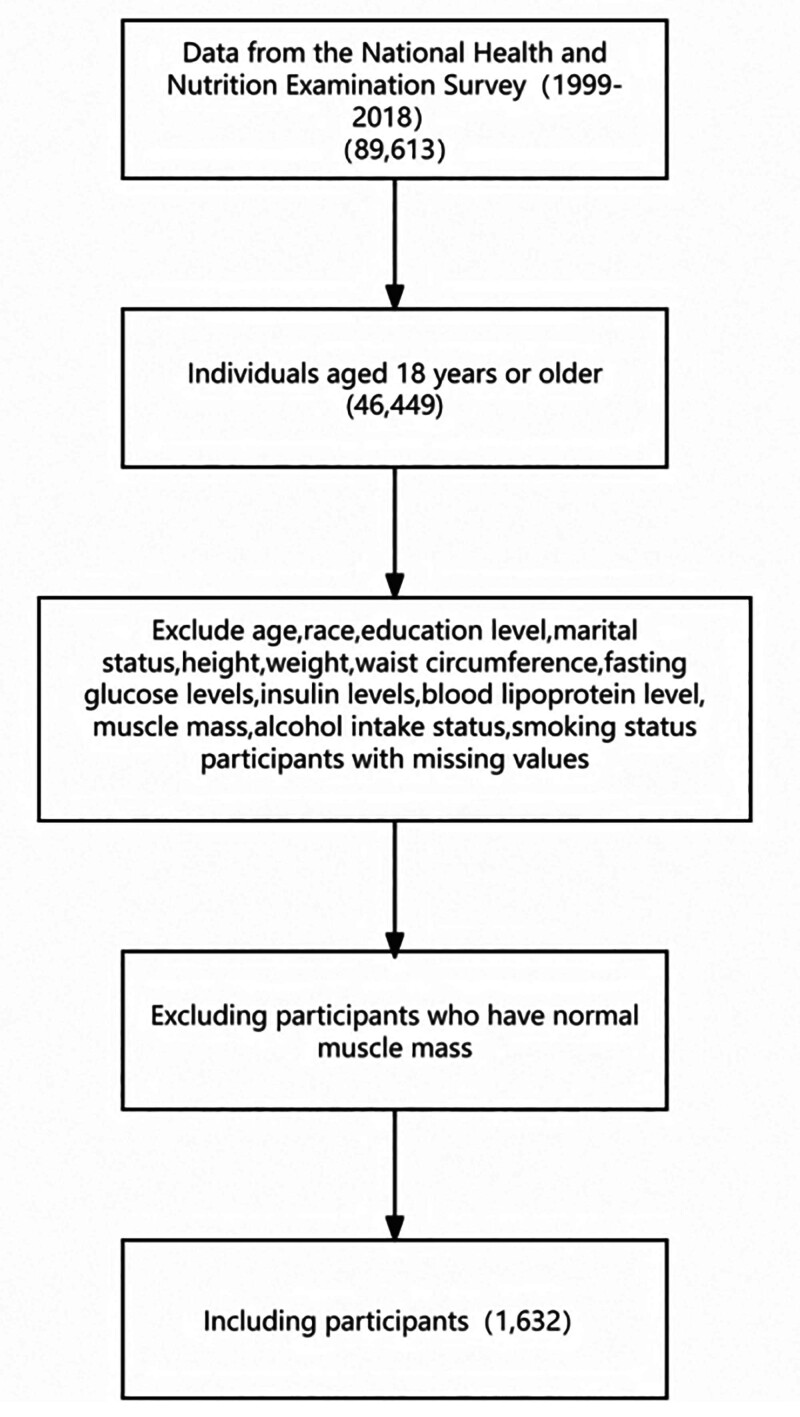
Flowchart.

The study initially enrolled 80,558 individuals from 9 consecutive cycles of NHANES who provided information on their age, gender, race, marital status, height, waist circumference, smoking status, weight, alcohol consumption, presence of hypertension and heart disease. Participants with missing data or under 18 years old were excluded. Additionally, individuals with normal muscle mass were also excluded. Ultimately, the analysis included a total of 1632 participants (Fig. 1).

### 2.2. Assessment of sarcopenia and diabetes mellitus

The evaluation was performed using the appendicular skeletal muscle mass index (ASMI), which is determined by dividing the skeletal muscle mass of the extremities by the square of an individual’s height (kg/m^2^). Dual-energy x-ray absorptiometry was used to measure the extremities. ASMI thresholds for inclusion in the study were <7.0 kg/m^2^ for men and <5.5 kg/m^2^ for women. Men with ASMI values below 7.0 kg/m^2^ and women with ASMI values below 5.5 kg/m^2^ were included in the study.^[12]^

Definition of diabetes mellitus: diabetes mellitus was defined as fasting glucose > 125 mg/dL, glycosylated hemoglobin > 6.5%, or self-reported diagnosis of new-onset diabetes mellitus or use of antidiabetic medications.

### 2.3. Assessment of METS-IR, BMI, TyG, TyG-WC, TyG-BMI and TG/HDL-C

In this study, the METS-IR index was used as the exposure variable and was calculated using the following formula: METS-IR = [Ln (2 × fasting glucose) + fasting triglycerides) × body mass index]/[Ln (high-density lipoprotein cholesterol)]. Fasting glucose and triglyceride levels were measured after an 8.5-hours fasting period using an automated biochemical analyzer. The body mass index (BMI) (kg/m^2^) was derived by dividing the weight (kg) of the individual by the square of their height (m). TyG = ln[TG (mg/dL) × FSG (mg/dL)/2]; TyG-WC = TyG × WC; TyG-BMI = TyG × BMI; triglyceride to high-density lipoprotein cholesterol ratio = TG/HDL-C.

### 2.4. Diabetes and multivariate logistic regression employing diverse indicators of insulin resistance

The dependent variable was diabetes status (coded as 1 = present, 0 = absent). Independent variables were selected through a systematic 2-stage process: univariate screening with a liberal threshold (*P* < .20) to identify potential covariates, followed by (2) multivariable logistic regression that included all clinically relevant variables regardless of their univariate significance, in accordance with Hosmer and Lemeshow’s purposeful selection method (Supplementary Material 1, Supplemental Digital Content, https://links.lww.com/MD/Q261). Continuous variables were assessed for linearity in the logit using fractional polynomials.

Model assumptions were rigorously verified, including:

1. Absence of multicollinearity (all variance inflation factors < 3; Supplementary Material 2, Supplemental Digital Content, https://links.lww.com/MD/Q261)
2. Adequate events per variable (EPV > 10)
3. Missing data handling (complete-case analysis with <5% missingness)

Categorical variables (e.g., race, hypertension, alcohol consumption) were coded as dummy variables, with the lowest category serving as the reference (e.g., Race: Mexican American as reference; Hypertension: No as reference; Alcohol consumption: No as reference). Continuous variables (e.g., age, METS-IR, triglyceride glucose index [TyG], triglyceride glucose index waist circumference [TyG-WC], triglyceride glucose index body mass index [TyG-BMI], and triglyceride to high-density lipoprotein cholesterol [TG/HDL-C]) were standardized using *z*-score transformation to facilitate effect size comparisons.

Multivariable binary logistic regression was performed using the forced entry method. The model included all clinically relevant covariates preselected based on existing literature. Dummy coding for polytomous categorical variables adhered to the following conventions:

For race (5 categories): 4 dummy variables were created, with Mexican American as the reference category.

For hypertension and alcohol consumption (2 categories): 1 dummy variable was created for each, with “No” as the reference category.

Reference categories were chosen based on clinical plausibility and sample size adequacy (>10% of the total sample). For categorical variables with more than 3 levels, the Šidák correction was applied to control the family-wise error rate. Model fit was assessed using the Hosmer–Lemeshow test (*P* = .32) and Nagelkerke *R*^2^ = 0.42. Sensitivity analyses conducted with different reference categories yielded consistent results.

### 2.5. Covariate adjustment

We employed a covariate adjustment model to investigate the impact of confounding variables on the association between METS-IR, TyG, TyG-WC, TyG-BMI, and TG/HDL-C with diabetes in individuals with sarcopenia. Independent variables in the logistic regression equation were selected based on a significance level of 0.2 after comparing the baseline table, with the diagnosis of diabetes considered as the dependent variable.The final set of covariate variables comprised age (in years), gender (male/female), race, education level, marital status, alcohol consumption (yes/no), smoking status (currently nonsmoker/currently smoker), as well as underlying medical conditions such as hypertension and coronary heart disease.

### 2.6. Statistical analysis

For normally distributed continuous variables, the mean (standard deviation, SD) was reported; for skewed variables, the median (interquartile range, IQR) was reported. Dichotomous variables were presented as frequencies and percentages. For comparisons of continuous variables, independent *t*-tests were used, while categorical variables were analyzed using Pearson chi-squared tests. The proportions (%) of baseline characteristics for categorical variables were compared using the chi-squared test. To assess the relationship between sarcopenia and diabetes, a multivariate logistic regression model was employed. Using the occurrence of diabetes as the dependent variable, variables with *P* < .2 in univariate and multivariate analyses and factors influencing clinical outcomes were included in the logistic regression model. Due to multicollinearity, fasting glucose level, BMI, HDL level, triglyceride level, as well as METS-IR, TyG, TyG-WC, TyG-BMI, and TG/HDL-C were excluded from the analysis. Odds ratios and 95% confidence intervals were calculated for METS-IR, BMI, TyG, TyG-WC, TyG-BMI, and TG/HDL-C in the study population. The statistical significance level was set at α = 0.05. Two multivariate adjusted models were constructed to account for confounding: model 1 adjusted for age and gender; model 2 adjusted for race, education level, marital status, alcohol consumption history, smoking history, hypertension history, and personal history of heart disease based on model 1.

The predictive ability of METS-IR, TyG, TyG-BMI, TyG-WC, TG/HDL-C, and TC/HDL-C for diabetes risk in individuals with or suspected of sarcopenia was assessed by area under the receiver operating characteristic curve (AUC) and optimal cutoffs were determined based on Youden index. A *P*-value < .05 was considered statistically significant.

All statistical analyses were performed using R version 4.3.2 (https://www.r-project.org/).

## 3. Result

### 3.1. Baseline characteristics table

Among the 1632 subjects included in this study with reduced muscle mass, 697 (43%) were male and 935 (57%) were female, with a mean age of 53 (45, 62) years. Among them, 184 (11.3%) had diabetes while 1448 (88.7%) did not have diabetes. Additionally, individuals with lower educational attainment, members of the Mexican–American population, and those with sarcopenia accompanied by hypertension exhibited higher prevalence rates of diabetes. However, no statistically significant differences were observed between the 2 groups regarding age, marital status, or smoking habits. METS-IR, BMI, TyG, TyG-WC, TyG-BMI, and TG/HDL-C levels were significantly higher (*P* < .001) in individuals with diabetes compared to those without (Table 1).

**Table 1:** Baseline clinical characteristics of participants by diabetes status participants by diabetes status.

	Total	Non-diabetes	Diabetes	*P*-value
	(n = 1632)	(n =1448)	(n = 184)	
Gender				.055
Male	697 (43%)	604 (42%)	93 (51%)	
Female	935 (57%)	844 (58%)	91 (49%)	
Age (yr)	53 (45, 62)	53 (45, 62)	54 (48.75, 62)	.096
Race				<.001
1. Mexican American	283 (17%)	228 (16%)	55 (30%)	
2. Other Hispanics/Latinos	115 (7%)	93 (6%)	22 (12%)	
3. Non-Hispanic White	791 (48%)	738 (51%)	53 (29%)	
4. African American	166 (10%)	150 (10%)	16 (9%)	
5. Other races-including multiracial	277 (17%)	239 (17%)	38 (21%)	
Education level				<.001
1. <9th grade	211 (13%)	169 (12%)	42 (23%)	
2. 9–11th grade (includes 12th grade with no diploma)	224 (14%)	188 (13%)	36 (20%)	
3. High school graduate/GED or equivalent	346 (21%)	314 (22%)	32 (17%)	
4. Some college or AA degree	449 (28%)	403 (28%)	46 (25%)	
5. College graduate or above	402 (25%)	374 (26%)	28 (15%)	
Marital status				.353
1. Married	936 (57%)	821 (57%)	115 (62%)	
2. Widowed	174 (11%)	159 (11%)	15 (8%)	
3. Divorced	234 (14%)	204 (14%)	30 (16%)	
4. Separated	59 (4%)	53 (4%)	6 (3%)	
5. Never married	147 (9%)	136 (9%)	11 (6%)	
6. Living with partner	82 (5%)	75 (5%)	7 (4%)	
Hypertension				.001
Yes	798 (49%)	737 (51%)	61 (33%)	
No	834 (51%)	711 (49%)	123 (67%)	
Heart disease				
Yes	1130 (69%)	1016 (70%)	114 (62%)	.029
No	502 (31%)	432 (30%)	70 (38%)	
Alcohol consumption				<.001
Yes	644 (39%)	540 (37%)	104 (57%)	
No	988 (61%)	908 (63%)	80 (43%)	
Smoking status				.132
Yes	1254 (77%)	1104 (76%)	150 (82%)	
No	378 (23%)	344 (24%)	34 (18%)	
High-density lipoprotein (mg/dL)	55 (45, 69)	56 (46, 69)	48 (40, 58)	<.001
Serum Triglycerides (mg/dL)	104 (73, 156)	101 (72, 149)	142.5 (96, 206)	<.001
BMI (kg/m^2^)	23.68 (21.23, 26.95)	23.5 (21.08, 26.49)	25.67 (22.93, 29.35)	<.001
Waist circumference (cm)	79 (37, 89.32)	78.75 (37, 89)	83 (37.22, 92.8)	.072
SMI (kg/m^2^)	6.02 (5.26, 6.93)	5.96 (5.25, 6.9)	6.45 (5.33, 7.88)	.06
Fasting glucose (mg/dL)	99.65 (92.9, 109)	98.35 (92, 106)	142.9 (111.97, 210.25)	<.001
METS-IR	30.7 (26.7,35.6)	30.2 (26.4,34.7)	35.7 (29.8,40.2)	<.001
TyG index	8.57 (8.18, 9.03)	8.52 (8.15, 8.93)	9.22 (8.74, 9.83)	<.001
TyG-WC	766.13 (670.01, 868.75)	751.45 (662.28, 854.18)	887.44 (795.73, 1004.98)	<.001
TyG-BMI	205.6 (177.99, 238.03)	202.3 (175.32, 232.24)	239.46 (209.72, 280.28)	<.001
TG/HDL	1.9 (1.14, 3.23)	1.81 (1.1, 2.98)	2.96 (1.75, 4.97)	<.001

Data are reported as no. (%) or mean (Q1, Q3). *P*-value were derived from χ^2^ test or Student’s test. *P*-value < .05.The *P*-value in the table indicates the statistical comparison between the diabetes group and the non-diabetes group.

BMI = body mass index, IR = insulin resistance, SMI = skeletal muscle mass index, MEST-IR = metabolic score for insulin resistance, TyG = triglyceride glucose, TyG-BMI = TyG-related to body mass index, TyG-WC = triglyceride glucose index waist circumference, TG/HDL-C = triglyceride to high-density lipoprotein cholesterol.

### 3.2. Association of METS-IR, BMI, TyG, TyG-WC, TyG-BMI and TG/HDL-C with the incidence of diabetes mellitus in people with sarcopenia

In both univariate and multivariate regression analyses, diabetes was designated as the dependent variable. Table 2 presents the associations between METS-IR, BMI, TyG, TyG-WC, TyG-BMI, and TG/HDL-C and diabetes incidence in the sarcopenia population within the regression models. The results indicate that METS-IR (OR: 1.088; 95% confidence interval (CI): 1.057–1.12), TyG (OR: 4.802; 95% CI: 3.355–6.873), TyG-WC (OR: 1.01; 95% CI: 1.008–1.013), TyG-BMI (OR: 1.019; 95% CI:1.013–1.013), and TG/HDL-C (OR: 1.101; 95% CI:1.03–1.177) were significantly positively correlated with diabetes incidence in sarcopenia patients (Table 2).

**Table 2:** Multivariate logistic regression between METS-IR, TyG, TyG-WC, TyG-BMI and TG/HDL-C and diabetes mellitus in a population with sarcopenia.

Characteristic	B	Standard error	Wald	*P*-value	OR (95% CI)	Overall *P*-value*
METS-IR						
METS-IR	0.084	0.015	32.735	<.001	1.088 (1.057, 1.12)	–
Age	0.002	0.007	0.061	.805	1.002 (0.988, 1.016)	–
Race						<.001
1. Mexican American	Ref					
2. Other Hispanics/Latinos	0.394	0.431	0.837	.36	1.483 (0.638, 3.448)	
3. Non-Hispanic White	−1.088	0.277	15.41	<.001	0.337 (0.196, 0.58)	
4. African American	−0.032	0.442	0.005	.943	0.969 (0.407, 2.304)	
5. Other races -including multiracial	0.406	0.354	1.314	.252	1.501 (0.75, 3.006)	
Hypertension						–
No	Ref					
Yes	0.806	0.271	8.838	.003	2.239 (1.316, 3.809)	
Alcohol consumption						–
No						
Yes	−0.676	0.23	8.662	.003	0.508 (0.324, 0.798)	
METS-IR	0.079	0.014	32.863	<.001	1.083 (1.054, 1.112)	
TyG						
TyG	1.569	0.183	73.531	<.001	4.802 (3.355, 6.873)	–
Age	−0.005	0.008	0.381	.537	0.995 (0.981, 1.01)	
Race						<.001
1. Mexican American	Ref					
2. Other Hispanics/Latinos	0.363	0.469	0.6	.439	0.995 (0.981, 1.01)	
3. Non-Hispanic White	−0.982	0.295	11.093	<.001	0.375 (0.21, 0.668)	
4. African American	0.574	0.468	1.5	.221	1.775 (0.709, 4.444)	
5. Other races -including multiracial	0.393	0.38	1.067	.302	1.481 (0.703, 3.119)	
Hypertension						–
No	Ref					
Yes	0.667	0.281	5.651	.017	1.949 (1.124, 3.378)	
Alcohol consumption						–
No	Ref					
Yes	−0.751	0.243	9.595	.002	0.472 (0.293, 0.759)	
Ty-GWC						
Ty-GWC	0.01	0.001	59.878	<.001	1.01 (1.008, 1.013)	–
Age	−0.005	0.007	0.43	.512	0.995 (0.981, 1.01)	–
Race						<.001
1. Mexican American	Ref					
2. Other Hispanics/Latinos	0.382	0.457	0.701	.403	1.465 (0.599, 3.586)	
3. Non-Hispanic White	−1.349	0.291	21.52	<.001	0.26 (0.147, 0.459)	
4. African American	0.139	0.447	0.096	.756	1.149 (0.479, 2.756)	
5. Other races-including multiracial	0.331	0.366	0.817	.366	1.392 (0.68, 2.851)	
Hypertension						–
No	Ref					
Yes	0.634	0.278	5.189	.023	1.885 (1.093, 3.253)	
Alcohol consumption						–
No	Ref					
Yes	−0.767	0.238	10.409	.001	0.465 (0.292, 0.74)	
Ty-GBMI						
Ty-GBMI	0.019	0.003	39.307	<.001	1.019 (1.013, 1.013)	–
Age	0	0.007	0.005	.946	1 (0.986, 1.015)	–
Race						<.001
1. Mexican American	Ref					
2. Other Hispanics/Latinos	0.463	0.437	1.12	.29	1.589 (0.674, 3.744)	
3. Non-Hispanic White	−1.061	0.279	14.465	<.001	0.346 (0.2, 0.598)	
4. African American	0.033	0.451	0.006	.941	1.034 (0.427, 2.502)	
5. Other races-including multiracial	0.478	0.358	1.79	.181	1.613 (0.801, 3.252)	
Hypertension						–
No	Ref					
Yes	0.829	0.272	9.268	.002	2.29 (1.343, 3.904)	
Alcohol consumption						–
No	Ref					
Yes	−0.709	0.232	9.367	.002	0.492 (0.312, 0.775)	
TG/HDL-C						
TG/HDL-C	0.096	0.034	8.013	.005	1.101 (1.03, 1.177)	–
Age	0.002	0.007	0.045	.832	1.002 (0.988, 1.016)	–
Race						<.001
1. Mexican American	Ref					
2. Other Hispanics/Latinos	0.309	0.422	0.535	.465	1.362 (0.595, 3.117)	
3. Non-Hispanic White	−1.207	0.274	19.369	<.001	0.299 (0.175, 0.512)	
4. African American	−0.092	0.427	0.046	.83	0.912 (0.395, 2.108)	
5. Other races -including multiracial	0.192	0.349	0.303	.582	1.212 (0.611, 2.403)	
Hypertension						–
No	Ref					
Yes	0.876	0.269	10.62	.001	2.402 (1.418, 4.067)	
Alcohol consumption						–
No	Ref					
Yes	−0.725	0.228	10.118	.001	0.485 (0.31, 0.757)	

CI = confidence interval, OR = odds ratio, METS-IR = metabolic score for insulin resistance, TyG = triglyceride glucose, TyG-BMI = triglyceride glucose index body mass index, TyG-WC = triglyceride glucose index waist circumference, TG/HDL-C = triglyceride to high-density lipoprotein cholesterol.

* Likelihood ratio test for entire variable.

### 3.3. Covariate adjustment

The result of covariate adjustment are shown in Table 3.We constructed 3 models; non-adjusted model; model 1 should be adjusted for: age (years) and gender; model 2 should be adjusted for: age (years); gender; education level; marital status; smoking status; alcohol intake, hypertension and heart disease. Following model adjustment, significant positive correlations (*P* < .05) were observed among METS-IR, TyG index (TyG), TyG-WC index (TyG-WC), TyG-BMI index (TyG-BMI), TG/HDL-C ratio and diabetes onset (Table 3).

**Table 3:** Covariate adjustment model between METS-IR, TyG, TyG-WC, TyG-BMI, TG/HDL-C and diabetes.

Characteristic	Crude OR (95% CI)	*P*-value	Model 1 OR (95% CI)	*P*-value	Model 2 OR (95% CI)	*P*-value
METS-IR	1.10 (1.07, 1.13)	<.001	1.09 (1.06, 1.12)	<.001	1.08 (1.05, 1.11)	<.001
TyG	5.25 (3.75, 7.36)	<.001	5.27 (3.74, 7.42)	<.001	4.81 (3.37, 6.85)	<.001
TyG-WC	1.01 (1.01, 1.01)	<.001	1.01 (1.01, 1.01)	<.001	1.00 (1.00, 1.01)	<.001
TyG-BMI	1.02 (1.01, 1.03)	<.001	1.02 (1.01, 1.03)	<.001	1.02 (1.01, 1.02)	<.001
TG/ HDL-C	1.13 (1.06, 1.22)	<.001	1.12 (1.04, 1.20)	<.001	1.10 (1.03, 1.17)	<.001

Data are presented as odds ratios, 95% confidence intervals, and *P*-value.

Crude model adjusts for: none.

Model 1 adjust for: age (yr); gender.

Model 2 adjust for: age (yr); gender; education level; marital status; smoking status; alcohol intake, hypertension and heart disease.

OR = odds ratio, CI = confidence intervals, MEST-IR = metabolic score for insulin resistance, TyG = triglyceride glucose, TyG-BMI = triglyceride glucose index body mass index, TyG-WC = triglyceride glucose index waist circumference, TG/HDL-C = triglyceride to high-density lipoprotein cholesterol.

### 3.4. METS-IR, TyG, TyG-WC, TyG-BMI, TG/ HDL-C and predictive value of diabetes mellitus

**Figure 2.**
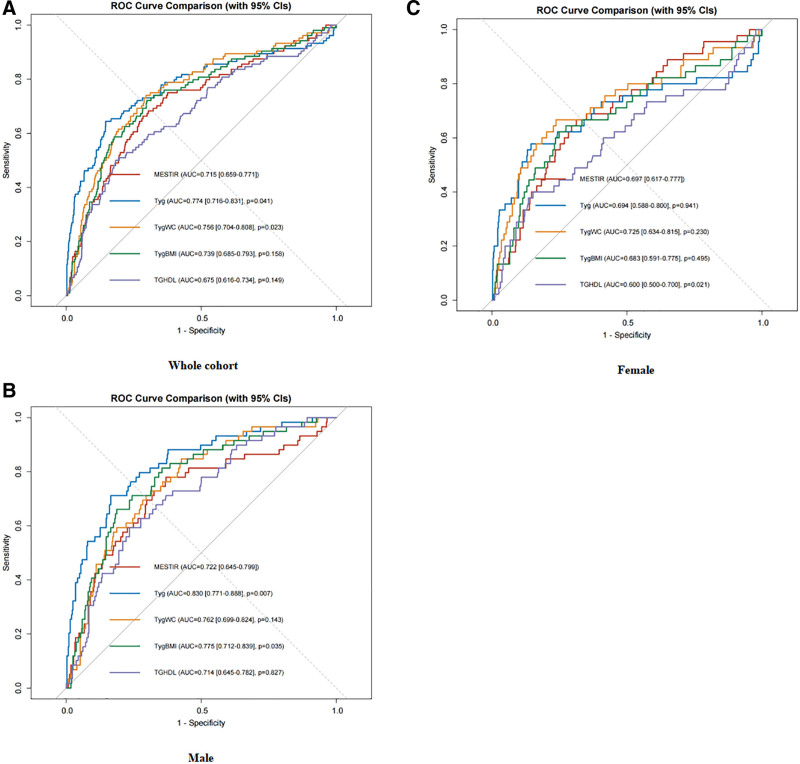
ROC curves for each index as predictors of diabetes. CI = confidence interval, ROC = receiver operating characteristic.

The receiver operating characteristic (ROC) curves of various indicators are depicted in Figure 2, while Table 4 presents the corresponding cutoff values and area under the curve (AUC) with sensitivity, specificity, and Youden index. In the overall population, TyG exhibits the highest AUC (0.77), with an optimal cutoff value of 9.15; followed by TyG-WC (AUC 0.76) with an optimal cutoff value of 793.89; whereas TG/HDL-C shows the lowest (AUC 0.68). Considering sex-specific variations in skeletal muscle mass index, subgroup analyses reveal that among males, TyG demonstrates the highest diagnostic value (AUC 0.830) at a cutoff value of 9.14, followed by TyG-BMI (AUC 0.78) at a cutoff value of 221.60; whereas among females, TyG-WC exhibits the highest diagnostic value (AUC 0.73) at a cutoff value of 772.70; followed by METS-IR (AUC 0.70) with an optimal cutoff of:32.88 (Table 4). Comparative analysis of ROC curves for METS-IR, TyG, TyG-WC, TyG-BMI, and TG/HDL-C revealed sex-specific differences in their discriminative ability. The TyG index demonstrated a significantly higher discriminative ability for diabetes mellitus in males (AUC: 0.82; 95% CI: 0.76–0.88) compared to females (AUC: 0.74; 95% CI: 0.68–0.80; DeLong’s test *P* = .013), indicating superior discriminative ability in male populations with sarcopenia (Fig. 2).

**Table 4:** Sensitivity, specificity, Youden index, cutoff points, and AUC (95% CI) for predicting the risk of diabetes by various indicators.

Characteristic	Cutoff point	AUC (95% CI)	Sensitivity	1-Specificity	Youden index
All					
METS-IR	34.66	0.72 (0.66, 0.77)	0.66	0.28	0.38
TyG	9.15	0.77 (0.72, 0.83)	0.64	0.15	0.45
TyG-WC	793.89	0.76 (0.70, 0.81)	0.74	0.30	0.45
TyG-BMI	206.97	0.74 (0.69, 0.79)	0.72	0.30	0.42
TG/HDL-C	3.37	0.68 (0.62, 0.73)	0.5	0.18	0.32
Male					
METS-IR	34.65	0.72 (0.65, 0.80)	0.78	0.37	0.41
TyG	9.14	0.83 (0.77, 0.89)	0.71	0.17	0.55
TyG-WC	793.89	0.76 (0.70,0.82)	0.85	0.43	0.42
TyG-BMI	221.60	0.78 (0.71, 0.84)	0.66	0.19	0.47
TG/ HDL-C	3.37	0.71 (0.65, 0.78)	0.59	0.23	0.36
Female					
METS-IR	32.88	0.70 (0.62, 0.78)	0.66	0.31	0.36
TyG	9.08	0.69 (0.59, 0.80)	0.58	0.15	0.43
TyG-WC	772.70	0.73 (0.63, 0.82)	0.67	0.24	0.43
TyG-BMI	209.96	0.68 (0.59, 0.78)	0.62	0.25	0.37
TG/ HDL-C	3.33	0.60 (0.50, 0.70)	0.4	0.15	0.25

AUC = area under the curve, CI = confidence intervals, OR = odd ratio, METS-IR = metabolic score for insulin resistance, TyG = triglyceride glucose, TyG-BMI = triglyceride glucose index body mass index, TyG-WC = triglyceride glucose index waist circumference, TG/HDL-C = triglyceride to high-density lipoprotein cholesterol.

## 4. Discussion

In recent years, the global prevalence of type 2 diabetes has increased dramatically. Sarcopenia, a major age-related complication, is bidirectionally associated with T2DM, and IR may represent a shared underlying mechanism.^[13]^ Numerous epidemiological studies have demonstrated associations between muscle loss and various IR markers. For example, research has revealed a significant positive correlation between the TyG index and muscle mass among American, Korean, and Chinese populations.^[14–16]^ In contrast, 1 cohort study reported a negative association between the TyG index and the incidence of sarcopenia in elderly Chinese individuals.^[17]^ Liu^[18]^conducted a cross-sectional study involving 4553 adults in the United States and found that METS-IR is a promising predictive marker for sarcopenia (AUC = 0.7217). Furthermore, studies have shown that METS-IR has a greater influence on the risk of sarcopenic obesity among individuals aged 65 years and older.^[19]^ Compared to the TyG index, Zhang^[20]^found that composite indices such as TyG-BMI and TyG-WC, which incorporate BMI and waist circumference (WC), demonstrate superior predictive performance for sarcopenia (AUC = 0.903, with sensitivity and specificity of 83.80% and 81.70%, respectively). A study based on the CHARLS database, which included 1427 elderly Chinese patients with diabetes, indicated that a higher TG/HDL-C ratio is linked to an increased risk of sarcopenia.^[21]^ Given that insulin resistance plays a central role in the pathogenesis of diabetes, multiple IR markers are closely related to both sarcopenia and the progression of diabetes. Although various insulin resistance indices are implicated in the onset of sarcopenia and diabetes, their utility in differentiating diabetes in individuals with sarcopenia remains controversial. Further investigation is warranted to validate these findings.

Building upon the aforementioned research, this study analyzed the metabolic characteristics of individuals with sarcopenia and comprehensively evaluated the discriminative ability of various insulin resistance (IR) markers – including METS-IR, TyG (TyG, TyG-WC, TyG-BMI), and TG/HDL-C – in assessing diabetes risk within the sarcopenic population.

Multivariate regression analysis revealed that all IR markers – METS-IR, TyG (TyG, TyG-WC, TyG-BMI), and TG/HDL-C – were significantly associated with diabetes risk (*P* < .05). After adjusting for covariates such as age, sex, education level, marital status, smoking and alcohol consumption status, and comorbidities (including hypertension and diabetes), the associations between METS-IR, TyG-related indices (TyG, TyG-WC, TyG-BMI), and diabetes risk remained statistically significant (*P* < .05), indicating that multiple IR markers are closely linked to the onset of diabetes in individuals with sarcopenia.

Among these markers, the TyG index showed the strongest association with diabetes risk (OR = 4.108, 95% CI: 2.942–5.735). ROC analysis further confirmed that the TyG index demonstrated the best discriminative performance for predicting diabetes onset in this patient group (AUC = 0.752). Notably, the TyG index exhibited superior discriminative efficacy in male patients (AUC = 0.830), which was significantly higher than that observed in female patients (AUC = 0.708, *P* < .05). This gender-based disparity may be attributed to the regulatory effects of sex hormones on lipid storage and metabolism, as well as the potential influence of androgens on insulin secretion,^[22]^ suggesting that the mechanisms underlying insulin resistance may differ between males and females.

In this study, the TyG index demonstrated superior predictive performance for diabetes compared to other IR markers, highlighting its distinct value in stratifying diabetes risk among individuals with sarcopenia. Although indicators such as METS-IR, TyG-WC, TyG-BMI, and TG/HDL-C were also associated with diabetes onset, their discriminative ability was lower than that of the TyG index. Notably, the TyG-WC indicator, which incorporates waist circumference, showed better discriminatory power specifically in female participants (AUC = 0.708). This suggests that gender differences may reflect more complex underlying mechanisms involved in the development of insulin resistance.

The findings support the hypothesis that insulin resistance remains a key contributor to diabetes development in individuals with reduced muscle mass. Potential underlying mechanisms include the following: during aging, hyperlipidemia triggers systemic inflammation and adipose tissue redistribution, leading to intramuscular lipid accumulation, which contributes to sarcopenia. On 1 hand, the accumulation of lipid metabolites such as diacylglycerol and ceramides within muscle cells can directly interfere with downstream insulin signaling pathways – particularly the translocation of glucose transporter 4 (GLUT4) – thereby inducing cellular insulin resistance. On the other hand, age-related mitochondrial dysfunction results in increased production of reactive oxygen species and localized inflammation, which activate stress signaling pathways including c-Jun N-terminal kinase, IκB kinase, protein kinase C, and p38 mitogen-activated protein kinase. These pathways inhibit insulin-PI3K-mTOR signaling, further impairing mitochondrial function and creating a self-perpetuating cycle involving sarcopenia, lipotoxicity, and muscle insulin resistance. Moreover, local insulin resistance reduces lipid uptake and elevates free fatty acid concentrations, thereby worsening local hyperlipidemia and inflammation. This promotes the secretion of inflammatory cytokines by both adipose and muscle tissues, leading to systemic inflammation, which exacerbates insulin resistance through multiple pathways and accelerates muscle atrophy. Consequently, these interrelated processes form a “metabolic aging cycle” that drives the progression of sarcopenic obesity and even cachexia.^[23,24]^

Our study demonstrates that among various insulin resistance-related indicators, the TyG index shows superior performance in identifying diabetes in individuals with sarcopenia. Notably, gender-specific differences were observed: the TyG index exhibited higher sensitivity for detecting diabetes in male sarcopenia patients, while the TyG-WC index demonstrated better performance in females. These findings provide important evidence for diabetes risk stratification and personalized management strategies in sarcopenic individuals, while also shedding light on potential pathways involved in the comorbidity of sarcopenia and metabolic disorders.

## 5. Limitations

However, this study has several limitations. First, the cross-sectional design prevents causal inferences from being drawn. Although these insulin resistance indices showed strong associations with prevalent diabetes, their predictive value for incident diabetes remains to be validated through prospective cohort studies involving serial measurements over time. Second, the absence of muscle function assessments – such as grip strength – may have underestimated the metabolic consequences associated with declining muscle strength. Third, the molecular mechanisms linking IR and sarcopenia require further experimental investigation. Additionally, the lack of data on mechanistic biomarkers – such as inflammatory cytokines and myokines – limits the depth of interpretation of the observed associations. Future research should incorporate longitudinal cohort designs and integrate multi-omics approaches to better elucidate the dynamic role of IR markers in the progression of sarcopenia.

## 6. Conclusions

This study aimed to evaluate the correlation between various indices of insulin resistance and the risk of diabetes mellitus within a sarcopenic population, as well as to compare the predictive capabilities of these indices for diabetes mellitus in this demographic.The findings of this research hold significant implications for healthcare professionals in their decision-making process when choosing appropriate indicators, enabling prompt identification of vulnerable populations, and implementing tailored interventions and therapies for these specific groups.Our results indicate that among the different insulin resistance indices assessed, we advocate for the use of the TyG index as a preferred predictor of diabetes mellitus in individuals with sarcopenia.

## Acknowledgments

Thank you to Xin Xia for the assistance provided during the article data statistics process.

## Author contributions

**Funding acquisition:** Yingying Zhu.

**Investigation:** Yingying Zhu, Jiabei He, Xin Li, Yunping Xu, Zongliang Yu, Kede Chi.

**Methodology:** Yingying Zhu, Jiabei He, Xin Li, Yunping Xu, Zongliang Yu, Kede Chi.

**Project administration:** Yingying Zhu, Jiabei He, Zongliang Yu.

**Resources:** Yingying Zhu, Xin Li, Yunping Xu.

**Software:** Yingying Zhu.

**Supervision:** Yingying Zhu, Kede Chi.

**Formal analysis:** Jiabei He.

## Supplementary Material


