# Supplementary material for: Comparing the effectiveness of different indicators of insulin resistance in predicting diabetes among adults with sarcopenia from the NHANES (1999–2018)
Source: Medicine (Baltimore). 2025 Oct 10;104(41):e45118. doi: 10.1097/MD.0000000000045118 (PMC12517797; doi:10.1097/MD.0000000000045118)
Supplement: Supplementary file 1 [file medi-104-e45118-s001.pdf]

**Supplementary Materials 1. Univariate and Multivariate Logistic Regression Analysis of Diabetes Risk Factors**

| Dependent: Diabetes | | unit | Coefficient (univariable) | Coefficient (multivariable) |
|---|---|---|---|---|
| Age(years) | [18.0,85.0] | mean(Q1,Q3) | 0.001 (-0.000 to 0.002, p=0.1780) | -0.001 (-0.002 to 0.000, p=0.1517) |
| Gender | Male | Ref | - | - |
| | Female | Mean (sd) | -0.047 (-0.084 to -0.010, p=0.0126) | 0.001 (-0.033 to 0.035, p=0.9606) |
| Race | [1.0,5.0] | Mean (sd) | -0.018 (-0.034 to -0.002, p=0.0260) | 0.012 (-0.002 to 0.027, p=0.0908) |
| Education Level | [1.0,5.0] | Mean (sd) | -0.031 (-0.044 to -0.017, p<0.0001) | -0.008 (-0.021 to 0.005, p=0.2132) |
| High Density Lipoprotein(mg/dL) | [18.0,173.0] | mean(Q1,Q3) | -0.002 (-0.003 to -0.001, p<0.0001) | -0.001 (-0.001 to 0.000, p=0.1592) |

| | | | | |
|---|---|---|---|---|
| Serum Triglycerides (mg/dL) | [23.0,1816.0] | mean(Q1,Q3) | 0.000 (0.000 to 0.001, p<0.0001) | 0.000 (-0.000 to 0.000, p=0.5513) |
| BMI(kg/m2) | [15.8,51.1] | mean(Q1,Q3) | 0.011 (0.006 to 0.017, p<0.0001) | 0.004 (-0.001 to 0.010, p=0.1255) |
| Waist circumference (cm) | [22.5,151.5] | mean(Q1,Q3) | 0.002 (0.001 to 0.003, p=0.0007) | -0.000 (-0.001 to 0.001, p=0.7288) |
| Fasting Glucose(mg/dL) | [57.0,442.0] | mean(Q1,Q3) | 0.005 (0.004 to 0.005, p<0.0001) | 0.004 (0.004 to 0.005, p<0.0001) |
| Hypertension | No | Ref | - | - |
| | Yes | | 0.071 (0.034 to 0.109, p=0.0002) | 0.046 (0.012 to 0.081, p=0.0085) |
| Alcohol consumption | No | Ref | - | - |

| | | | | |
|---|---|---|---|---|
| | Yes | | -0.086 (-0.123 to -0.049, p<0.0001) | -0.045 (-0.078 to -0.013, p=0.0064) |
| Smoking status | No | Ref | - | - |
| | Yes | | -0.046 (-0.089 to -0.003, p=0.0372) | -0.024 (-0.062 to 0.013, p=0.2070) |

**Data are reported as no. (%) or mean(Q1,Q3).P-value were derived from ｘ2 test or Student' s test.P- value ＜0.05.The P-value in the table indicates the statistical comparison between the diabetes group and the non-diabetes group.BMI,Body Mass Index.**

**Supplementary Materials 2. Multicollinearity Assessment Using Variance Inflation Factors (VIF) in the Multivariate Model**

| Dependent: Diabetes | Characteristic | B | Standard Error | Wald | P-value | VIF |
|---|---|---|---|---|---|---|
| | MESTIR | 0.008 | 0.001 | 0.19 | <.001 | 1.153 |
| | Gender | -0.029 | 0.019 | -0.047 | 0.132 | 1.091 |
| | Age(years) | -0.001 | 0.001 | -0.044 | 0.188 | 1.22 |
| | Race | 0.002 | 0.008 | 0.006 | 0.852 | 1.198 |
| | Education Level | -0.019 | 0.007 | -0.085 | 0.013 | 1.281 |
| | Marital Status | -0.005 | 0.006 | -0.022 | 0.474 | 1.054 |
| | Hypertension | 0.037 | 0.021 | 0.06 | 0.074 | 1.261 |
| | Alcohol consumption | -0.059 | 0.019 | -0.097 | 0.002 | 1.097 |
| | Smoking status | -0.043 | 0.022 | -0.06 | 0.055 | 1.097 |
| | Heart Disease | -0.009 | 0.021 | -0.014 | 0.647 | 1.104 |

**A:MESTIR**

| Dependent: Diabetes | Characteristic | B | Standard Error | Wald | P-value | VIF |
|---|---|---|---|---|---|---|
| | TyG index | 0.152 | 0.014 | 0.327 | <0.001 | 1.153 |
| | Gender | -0.034 | 0.018 | -0.055 | 0.065 | 1.064 |
| | Age(years) | -0.001 | 0.001 | -0.076 | 0.018 | 1.237 |
| | Race | 0 | 0.008 | 0.001 | 0.968 | 1.181 |
| | Education Level | -0.016 | 0.007 | -0.071 | 0.032 | 1.282 |
| | Marital Status | -0.001 | 0.006 | -0.006 | 0.828 | 1.058 |
| | Hypertension | 0.024 | 0.02 | 0.039 | 0.233 | 1.266 |
| | Alcohol consumption | -0.056 | 0.018 | -0.092 | 0.003 | 1.097 |

| | | | | | | |
|---|---|---|---|---|---|---|
| | Smoking status | -0.056 | 0.021 | -0.079 | 0.009 | 1.089 |
| | Heart Disease | -0.022 | 0.02 | -0.034 | 0.265 | 1.11 |

**B:TyG index**

| **Dependent: Diabetes** | **Characteristic** | **B** | **Standard Error** | **Wald** | **P-value** | **VIF** |
|---|---|---|---|---|---|---|
| | TyG-WC | 0.001 | 0 | 0.245 | <0.001 | 1.216 |
| | Gender | -0.013 | 0.019 | -0.022 | 0.489 | 1.125 |
| | Age(years) | -0.001 | 0.001 | -0.066 | 0.048 | 1.239 |
| | Race | 0.001 | 0.008 | 0.003 | 0.919 | 1.187 |
| | Education Level | -0.019 | 0.007 | -0.084 | 0.012 | 1.277 |
| | Marital Status | -0.005 | 0.006 | -0.025 | 0.407 | 1.052 |
| | Hypertension | 0.032 | 0.02 | 0.053 | 0.116 | 1.264 |

| | | | | | | |
|---|---|---|---|---|---|---|
| | Alcohol consumption | -0.062 | 0.019 | -0.103 | 0.001 | 1.094 |
| | Smoking status | -0.047 | 0.022 | -0.067 | 0.032 | 1.091 |
| | Heart Disease | -0.019 | 0.02 | -0.029 | 0.35 | 1.113 |

**C:TyG-WC**

| **Dependent: Diabetes** | **Characteristic** | **B** | **Standard Error** | **Wald** | **P-value** | **VIF** |
|---|---|---|---|---|---|---|
| | TyG-BMI | 0.002 | 0 | 0.217 | <0.001 | 1.134 |
| | Gender | -0.038 | 0.019 | -0.063 | 0.04 | 1.064 |
| | Age(years) | -0.001 | 0.001 | -0.055 | 0.094 | 1.229 |
| | Race | 0.003 | 0.008 | 0.011 | 0.728 | 1.2 |
| | Education Level | -0.019 | 0.007 | -0.086 | 0.011 | 1.277 |
| | Marital Status | -0.004 | 0.006 | -0.02 | 0.508 | 1.055 |

| | | | | | | |
|---|---|---|---|---|---|---|
| | Hypertension | 0.036 | 0.021 | 0.058 | 0.083 | 1.26 |
| | Alcohol consumption | -0.063 | 0.019 | -0.104 | <0.001 | 1.094 |
| | Smoking status | -0.039 | 0.022 | -0.055 | 0.08 | 1.099 |
| | Heart Disease | -0.012 | 0.02 | -0.019 | 0.545 | 1.106 |

**D:TyG-BMI**

| **Dependent: Diabetes** | **Characteristic** | **B** | **Standard Error** | **Wald** | **P-value** | **VIF** |
|---|---|---|---|---|---|---|
| | TG/HDL | 0.013 | 0.003 | 0.118 | <.001 | 1.069 |
| | Gender | -0.042 | 0.019 | -0.069 | 0.028 | 1.067 |
| | Age(years) | -0.001 | 0.001 | -0.041 | 0.224 | 1.222 |
| | Race | -0.005 | 0.008 | -0.019 | 0.555 | 1.176 |
| | Education Level | -0.02 | 0.008 | -0.093 | 0.007 | 1.277 |

| | | | | | |
|---|---|---|---|---|---|
| Marital Status | -0.006 | 0.006 | -0.028 | 0.376 | 1.053 |
| Hypertension | 0.045 | 0.021 | 0.074 | 0.03 | 1.251 |
| Alcohol consumption | -0.063 | 0.019 | -0.104 | 0.001 | 1.095 |
| Smoking status | -0.059 | 0.022 | -0.083 | 0.009 | 1.093 |
| Heart Disease | -0.01 | 0.021 | -0.016 | 0.617 | 1.11 |

**E:TG/HDL**
